# Neglected errors in ligament reconstruction surgery may increase graft-tunnel mismatch: A biomechanical study

**DOI:** 10.1371/journal.pone.0309146

**Published:** 2024-09-12

**Authors:** Hongwei Xu, Weichao Jiang, Songying Du, Honggang Zhu, Rui Sun, Xuejian Bai, Bo Wu, Qun Li, Da Huo, Huaiyu Yang

**Affiliations:** 1 Department of Orthopedics, General Hospital of Fuxin Mining Industry Group, Liaoning Health Industry Group, Fuxin, China; 2 Department of Orthopedics, Jilin Yan’an Hospital, Changchun, China; Menoufia University, EGYPT

## Abstract

Tunnel widening is a frequent problem following arthroscopic ligament reconstruction surgery that may primarily arise from a graft-tunnel mismatch caused by errors in surgical instruments and methods. The present study aimed to observe the influence of current surgical instruments and methods on graft-tunnel matching. We established an in vitro model using porcine Achilles tendons and tibias, and compared traditional surgical instruments (control group) with custom instruments (experimental group). Graft measurements, bone-tunnel creation, and measurements of the maximum pullout force of the graft from the bone tunnel were performed. Results indicated that the measuring gauge developed by our research group (capable of accurate measurement of graft diameters) may mitigate errors arising from graft-diameter measurement using traditional measuring cylinders. Therefore, errors caused by current surgical instruments and surgical methods led to an increase in graft-tunnel mismatches. The degree of mismatch was greater at the tibial end than at the femoral end.

## 1 Introduction

The tunnel-widening phenomenon has been regarded as the primary postoperative complication after ligament reconstruction since its first description in the 1990s [1–4]. Tunnel widening is defined as a widening of more than 2 mm in the bone tunnel within 2 years postoperatively [5]. It may result in poor graft and bone healing and post-reconstruction ligament laxity, thereby affecting secondary ligament reconstruction [1,6–8]. Researchers have devoted years of efforts to the exploration of the causes and solutions of tunnel widening, but the mechanisms of this phenomenon remain unclear. It is known that tunnel widening is influenced by multiple types of factors, including mechanical, biological, biomechanical, and surgical factors [1,4,6,9–12]. In particular, mechanical factors primarily give rise to the bungee effect and windshield-wiper effect [7,13–16]. The windshield-wiper effect refers to transverse motion of the graft perpendicular to the axis of the tunnel, whereas the bungee effect refers to longitudinal motion of the graft along the axis of the tunnel. The occurrence of the windshield-wiper and bungee effects may cause collisions between the graft and the bone tunnel, which may disrupt graft healing within the bone tunnel and contribute to tunnel widening. Therefore, mitigation of the bungee and windshield-wiper effects has become a pressing task for researchers.

In clinical surgeries, our research group has observed that graft-tunnel mismatches caused by current surgical instruments and methods may be the primary reason for the bungee and windshield-wiper effects. However, a scarcity of relevant studies currently exists in the literature. The present study utilized porcine Achilles tendons and porcine tibias for the establishment of an in vitro model. First, graft and tunnel diameters and the peak pullout force of the graft from the bone tunnel were measured using traditional surgical instruments. Subsequently, graft-diameter measurements were conducted with a self-designed measuring gauge, bone tunnels were created with custom-made drill bits and subjected to diameter measurements, and the peak pullout force of the graft from the bone tunnel was measured. The degree of graft-tunnel match was validated using the graft diameter, bone-tunnel diameter, and the maximum pullout force of the graft from the bone tunnel. Measurements obtained with traditional surgical instruments were assigned to the traditional (control) group, whereas measurements obtained with the modified instruments were assigned to the modified (experimental) group. By comparing data between the traditional and modified groups, we found that the use of traditional surgical instruments and methods was the main cause of increased graft-tunnel mismatches.

## 2 Data and methods

This study was approved by the ethics committee of our institution. Equipment used in the study included the following: traditional surgical instruments (Smith & Nephew); a measuring gauge developed by our research group (Ruipai Medical Technology (Changzhou) Co., Ltd.); custom-made drill bits (Shanghai Weigong Metal Products Co., Ltd.); pin gauges for measurement of bone-tunnel diameters (Shenzhen Lifeng Hardware Co., Ltd.); and a force gauge for measuring pulling force (Shence Intelligent Technology (Shenzhen) Co., Ltd.). The photographs shown in this article were taken with a Huawei Nova 7 SE mobile phone.

### 2.1 Operating principles of the self-designed measuring gauge graft description and development of the measuring gauge

**Fig 1:**
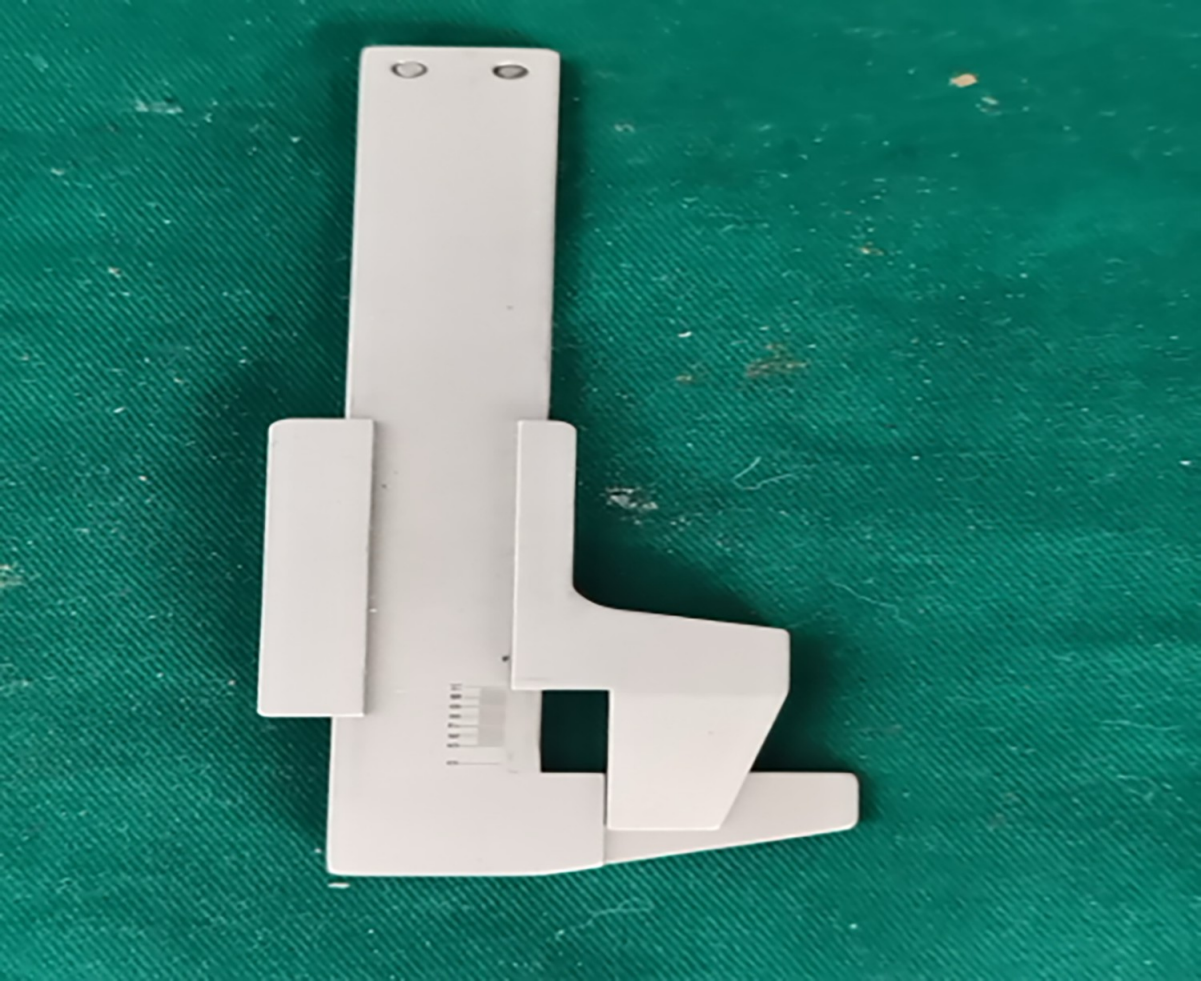
Graft diameter measuring gauge by our research group.

Grafts are tissue structures with contractile and elastic properties. The variability of a graft increases when it is compressed, but currently, no measuring gauges that enable accurate measurement of graft diameters are available in clinical practice. Therefore, our research group developed a measuring gauge (Fig 1) based on the mathematical principle that the perimeter of the cross-section of the graft remains unchanged regardless of whether the compressed cross-section is rectangular or circular. Therefore, the perimeter = π × diameter. By measuring the cross-sectional perimeter of the graft using our self-developed measuring gauge, the graft diameter can be calculated.

### 2.2 Preparation of the measuring gauge

Currently, grafts used in anterior and posterior cruciate ligament reconstruction surgeries in clinical practice have a diameter of 10 mm or less. The measurement gauge designed by our research group had a fixed width of 10 mm and variable length. By harnessing the elastic properties of the graft, the measuring gauge could be adjusted to enable complete filling of the gauge by the compressed graft. The diameter of the graft could then be calculated based on the relationship between perimeter and diameter.

### 2.3 Experimental methods and samples

Porcine tibias and Achilles tendons were used as experimental materials. The size of the porcine tibias and biomechanical properties such as bone density were similar to those of young humans, and the porcine Achilles tendons also exhibited material properties similar to those of humans [17–20]. A total of 35 porcine Achilles tendons and 35 porcine tibias were purchased from a slaughterhouse. Specimens were stored at -20°C and thawed at room temperature for 24 h before use.

### 2.4 Preparation and measurement of grafts

**Fig 2:**
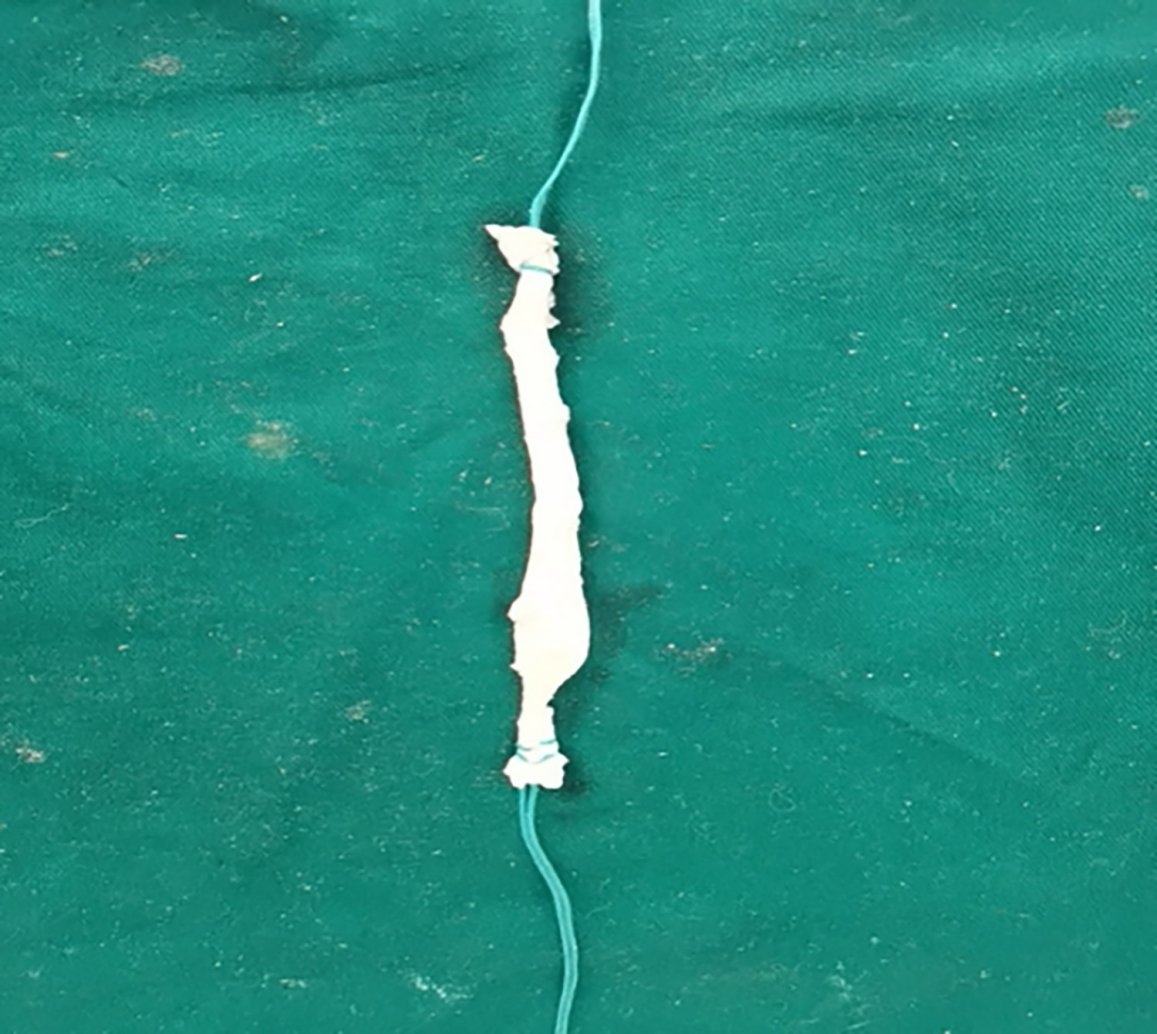
Grafts prepared using porcine developed Achilles tendons.

**Fig 3:**
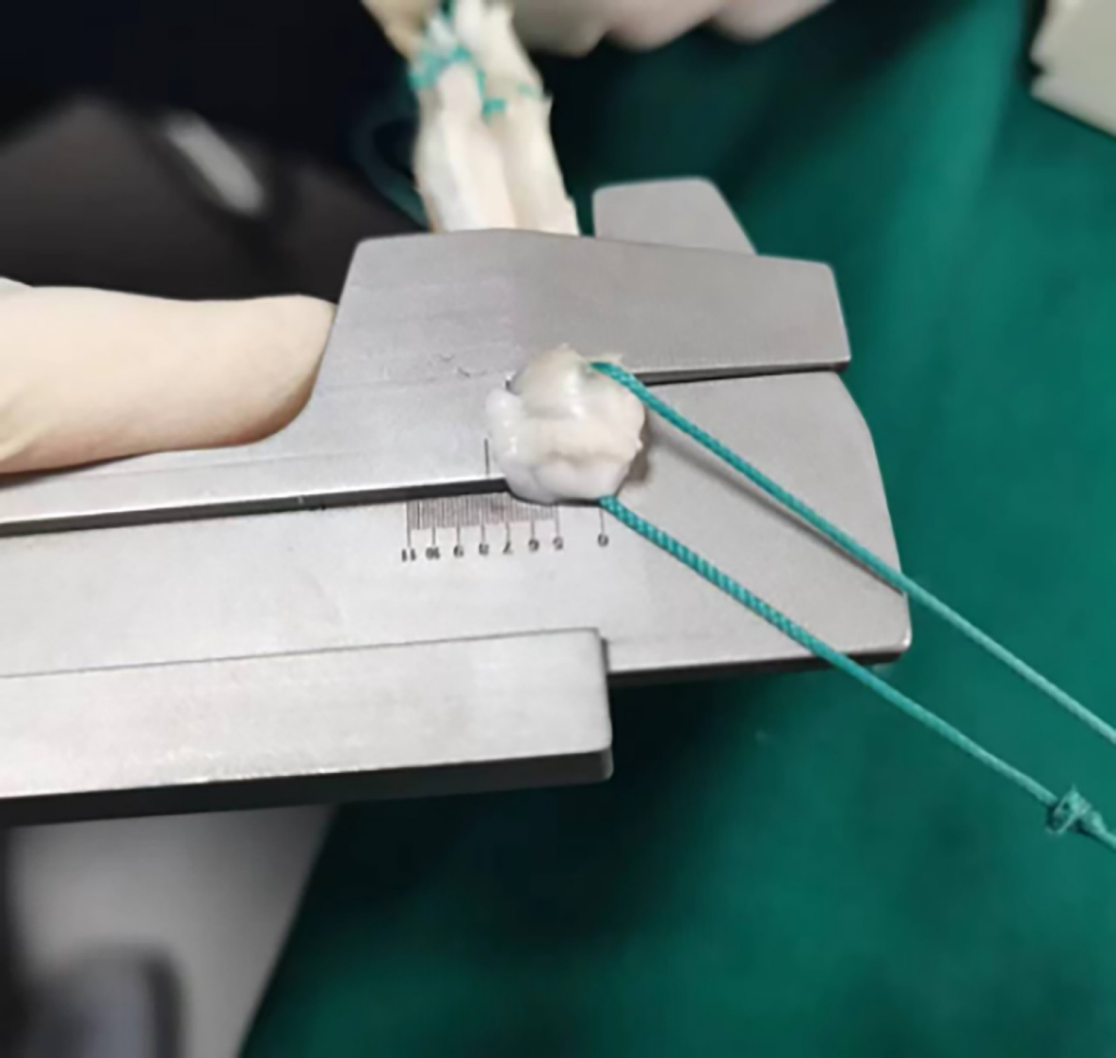
Measurement of graft diameter using the self-developed measuring gauge.

Porcine Achilles tendons were excised, subjected to removal of surface muscle tissue, and woven into grafts (Fig 2). The diameter of each prepared graft was subsequently measured by both the traditional and modified methods: (1) traditional-the smallest diameter of the measuring cylinder that allowed smooth passage of the graft was taken as the graft diameter; and (2) modified-the graft diameter was directly measured using the measuring gauge developed by our research group (Fig 3).

### 2.5 Bone-tunnel creation and pulling-force measurement

**Fig 4:**
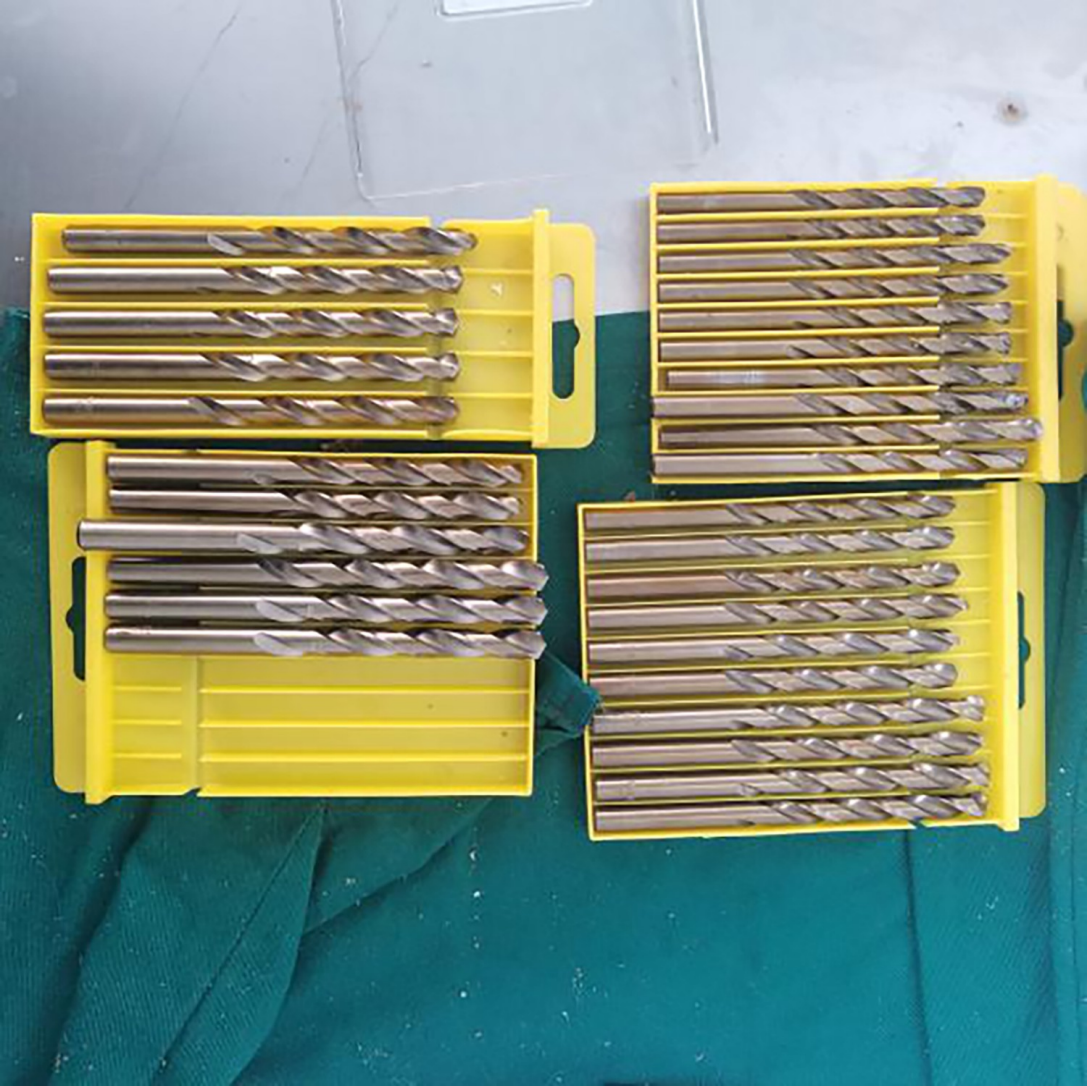
Custom-made drill bits with diameter intervals of 0.1 mm used in this study.

**Fig 5:**
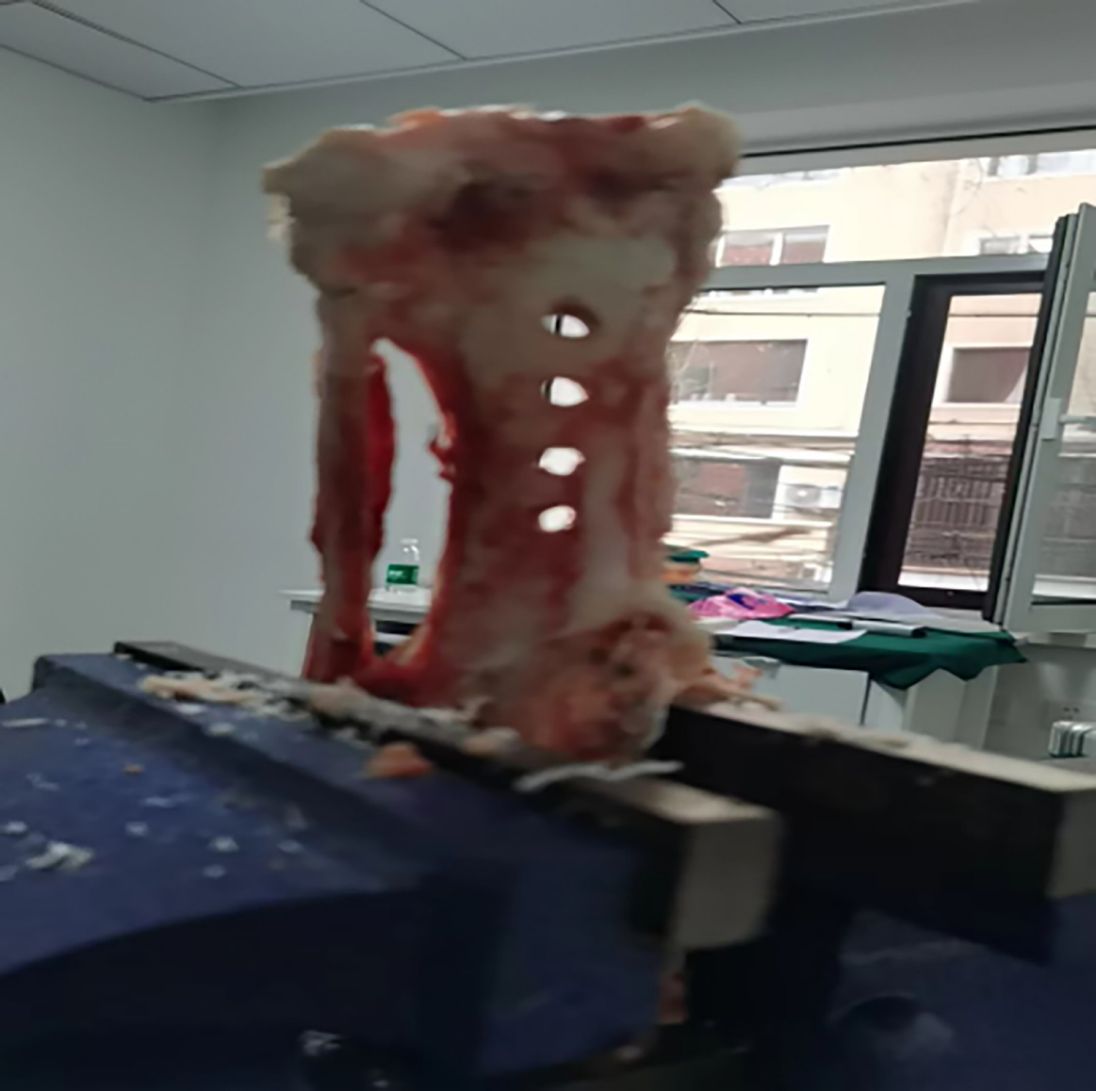
According to the measurement results of the diameter of the graft, the proximal and distal bone tunnels are made, respectively.

**Fig 6:**
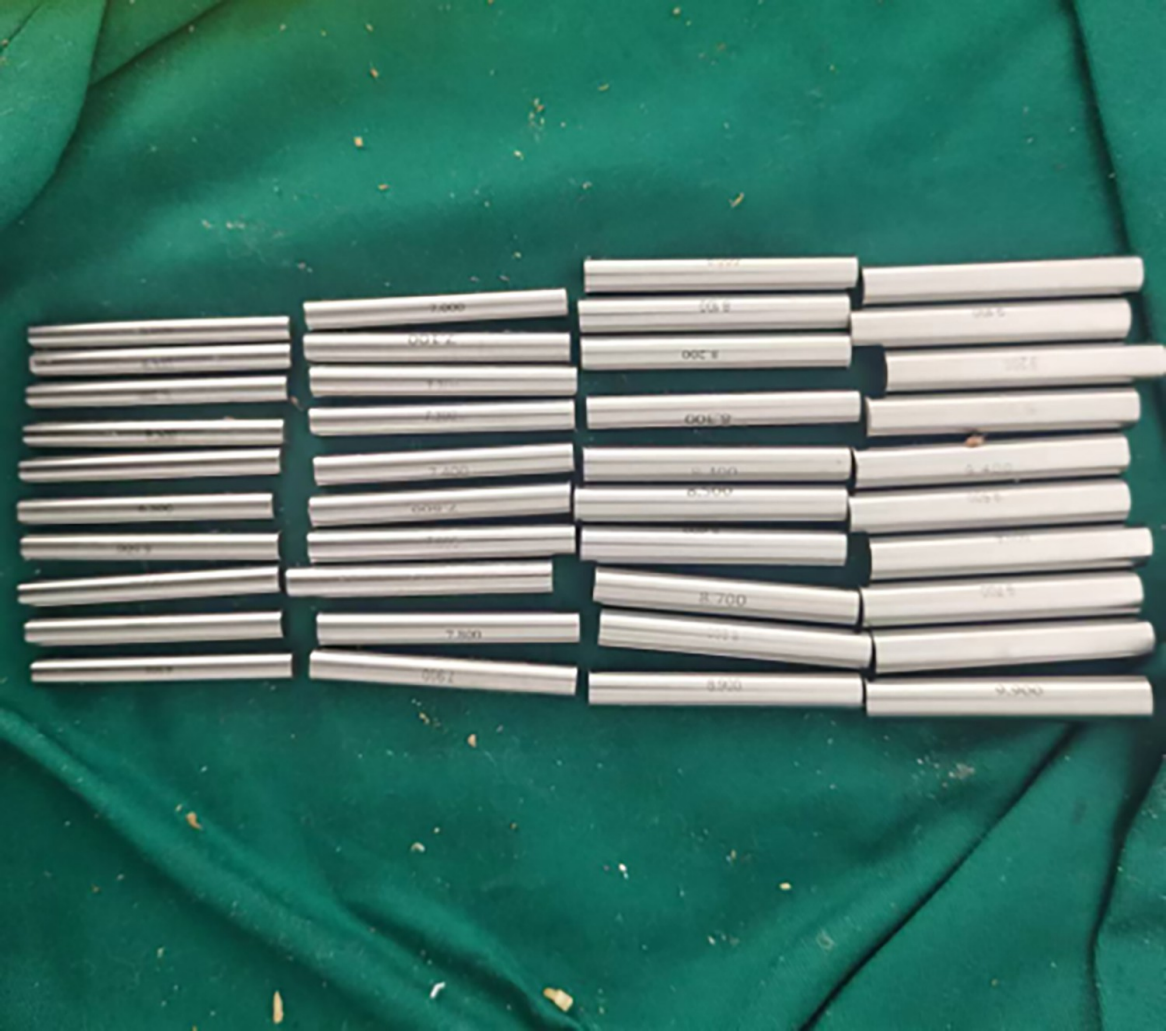
Pin gauges used for measuring bone tunnel diameter.

**Fig 7:**
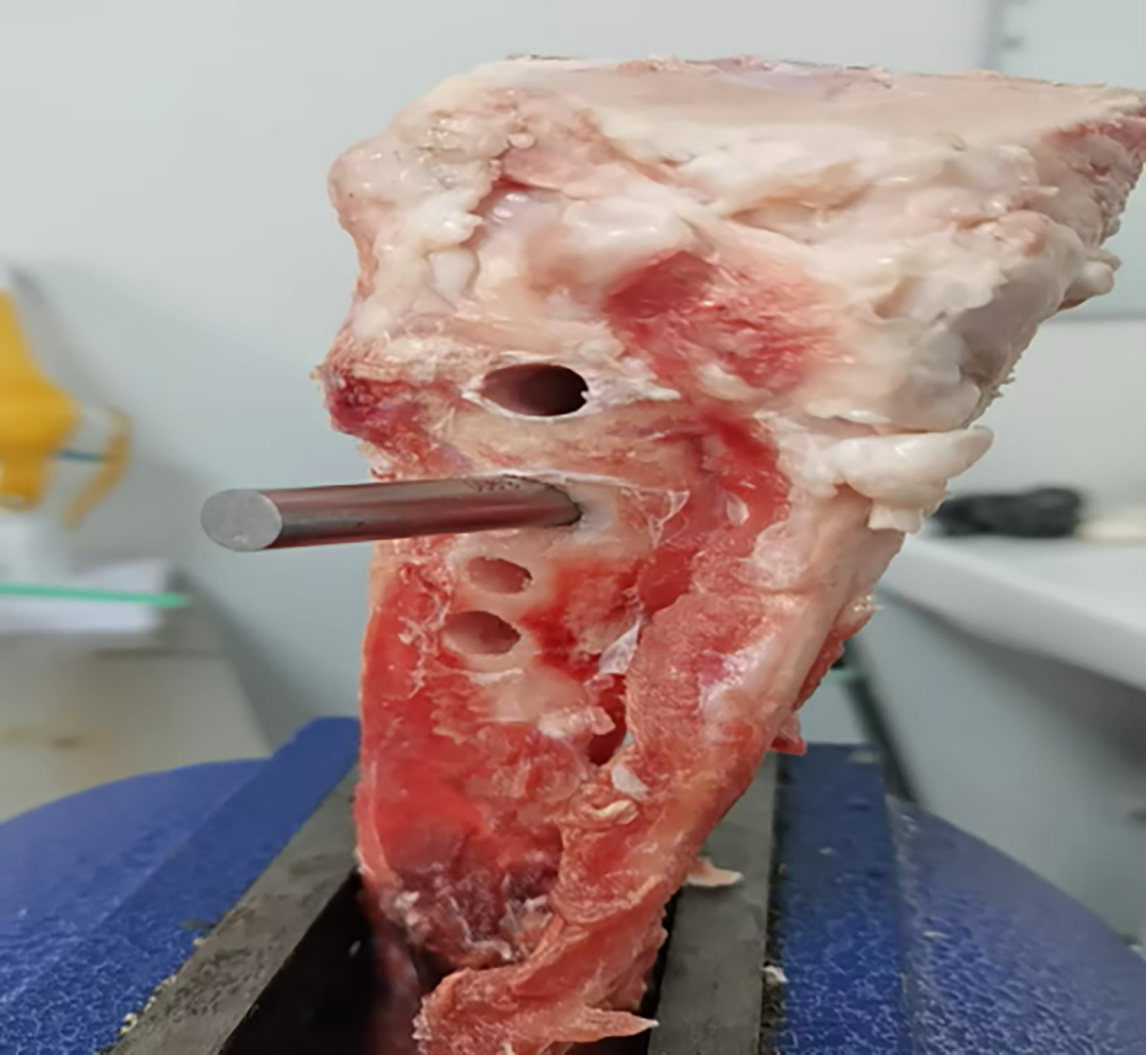
Measurement of bone tunnel diameter with a pin gauge.

**Fig 8:**
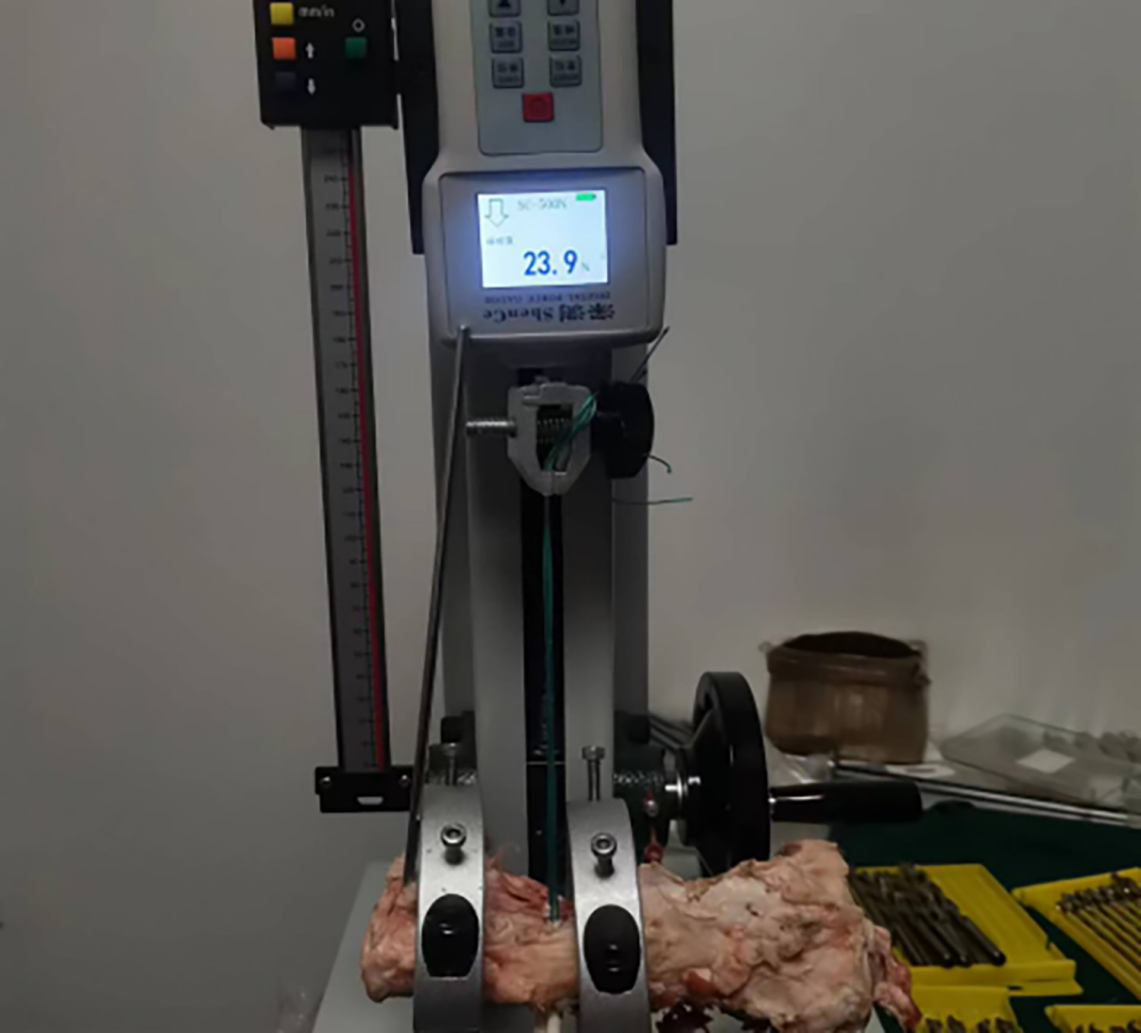
Porcine tibia mounted onto the force gauge with the graft, bone tunnels, and pulling force maintained in a vertical direction.

Based on the graft-diameter measurements, bone tunnels were created using drill bits with corresponding diameters. The drill bits used in the traditional group had diameters of 6, 7, 8, and 9 mm, whereas those of the modified group were custom-made and had diameter intervals of 0.1 mm (Fig 4), which allowed close matching with the graft-diameter measurements. A single porcine tibia was used to create four bone tunnels based on the measurements, which were separately labeled as the traditional proximal bone tunnel, traditional distal bone tunnel, modified proximalbone tunnel, and modified distal bone tunnel (Fig 5). Soft tissue near the bone tunnels and intraosseous tissue were both removed to avoid affecting the pulling-force measurements. The diameters of the drilled bone tunnels were measured using pin gauges and recorded (Figs 6 and 7). Each porcine tibia was mounted onto the force gauge with the graft, bone tunnels, and pulling force maintained in a vertical direction (Fig 8). For the traditional group, 2 cm of the proximal end of the woven graft was pulled into the proximal bone tunnel, and 2 cm of the distal end was pulled into the distal bone tunnel. Next, the peak pulling force was measured on the force gauge and recorded. The modified group measurements were obtained in a similar fashion; 2 cm of the proximal end of the woven graft was pulled into the proximal bone tunnel, and 2 cm of the distal end was pulled into the distal bone tunnel. Finally, the peak pulling force was measured on the force gauge and recorded.

**Fig 9:**
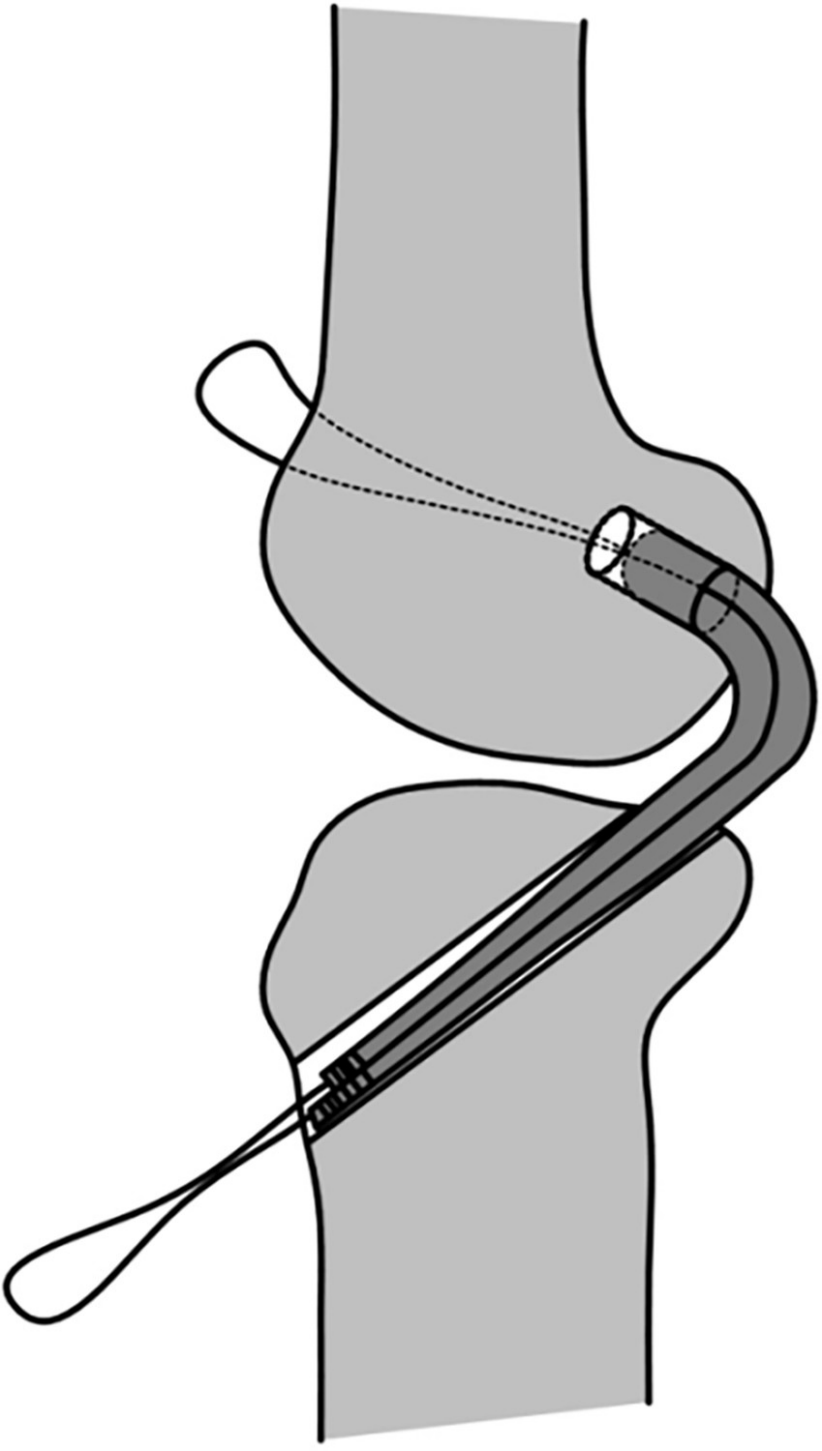
Passage of entire graft through the tibial tunnel.

**Fig 10:**
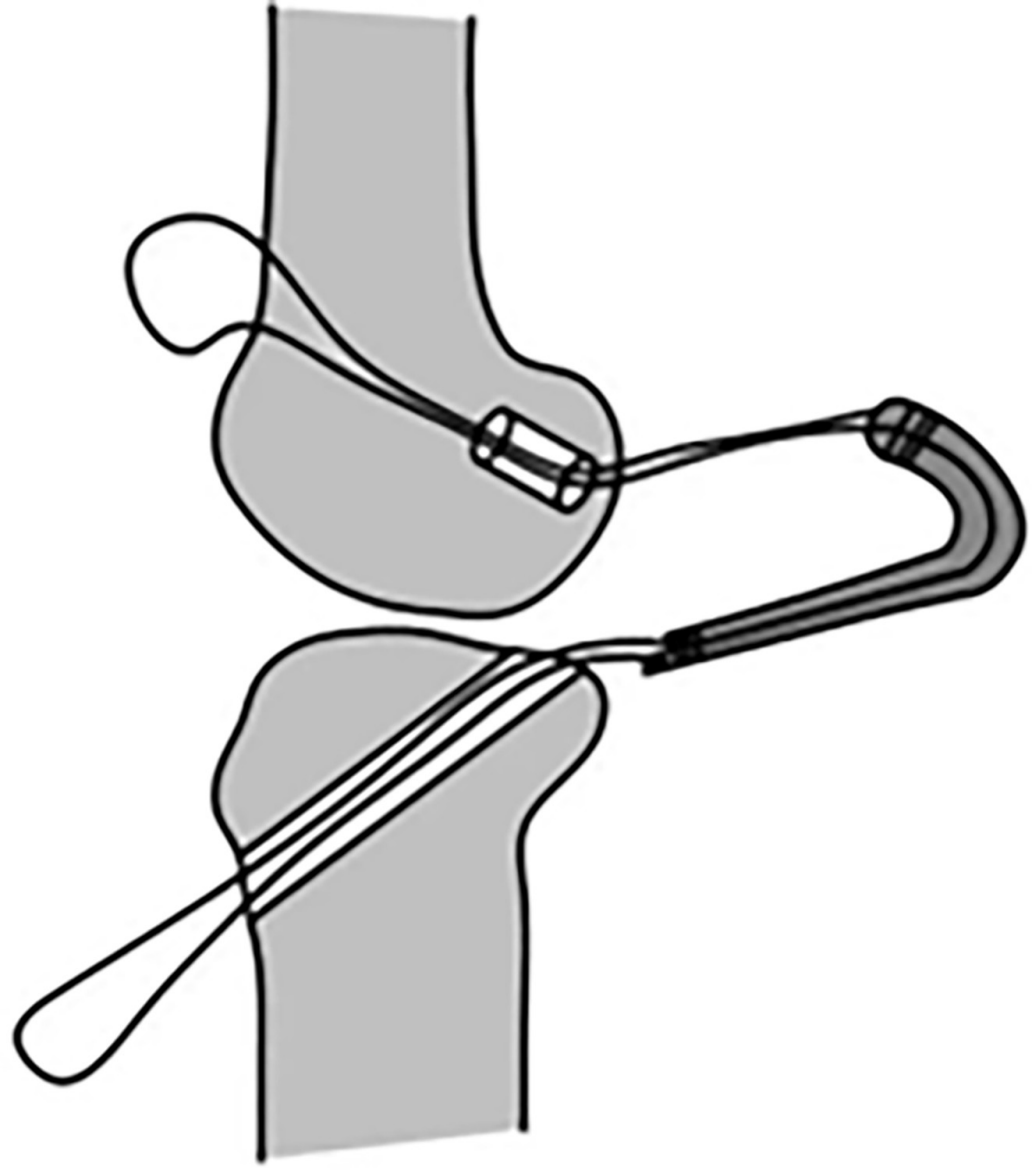
Passage of entire graft through the tibial tunnel was unnecessary for the control group.

**Fig 11:**
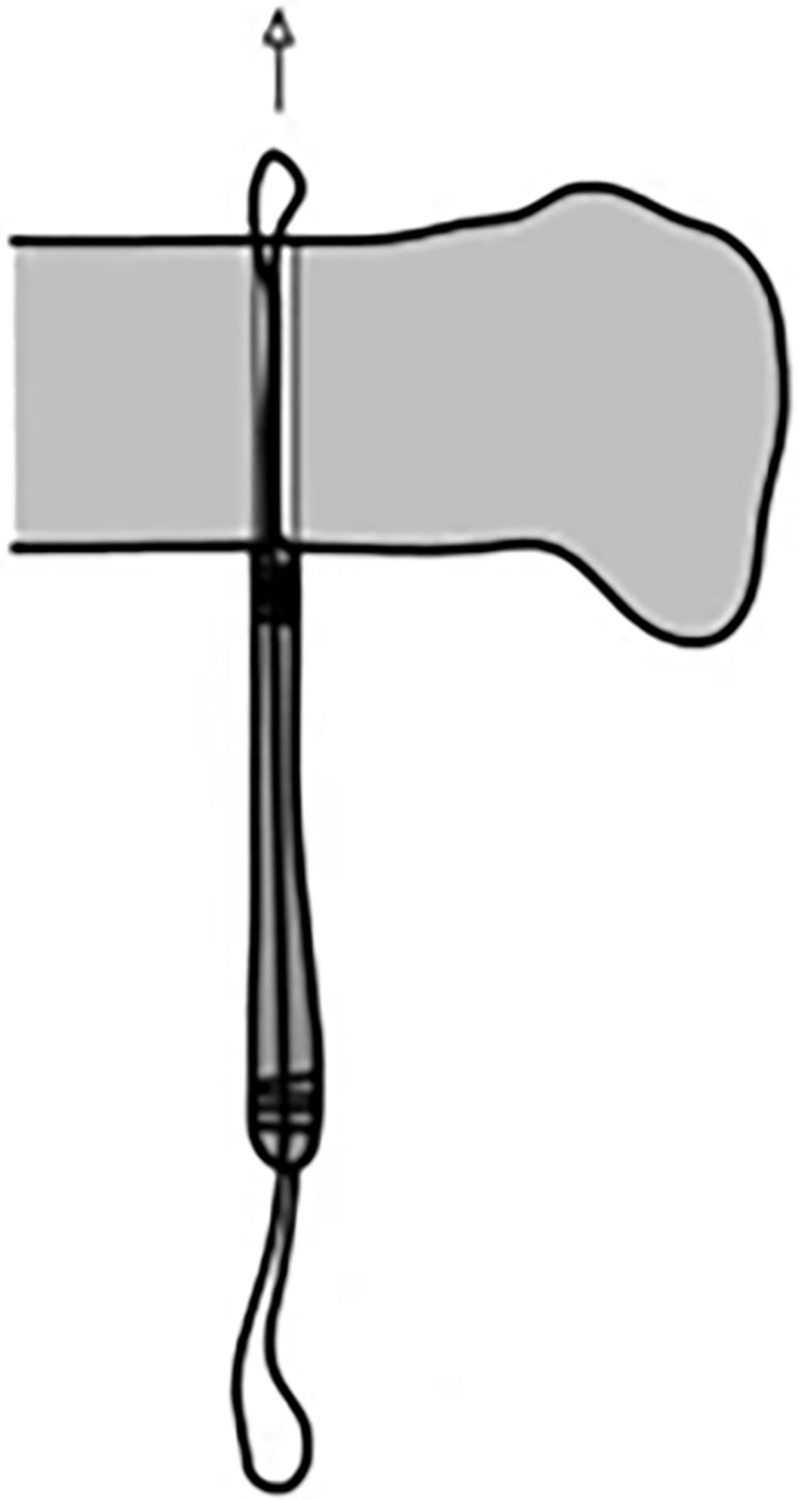
For the control group, the distal 2 cm of the graft was inserted into the bone tunnel before measurement of the peak pulling force during retrograde pulling of the graft.

The traditional surgical method requires complete passage of the proximal end and middle portion of the graft through the tibial tunnel. Subsequently, the proximal 2 cm of the graft is pulled into the femoral tunnel and secured, and the distal 2 cm is secured onto the tibial tunnel. Given that the entire graft is passed through the bone tunnel in the traditional surgical method (Fig 9), the distal graft diameter measured by the measuring cylinder is equivalent to the maximum diameter of the graft. In the control group, the surgical method involves placement of the graft into the joint cavity through the eye of the knee. The proximal 2 cm of the graft is then directly pulled into the proximal femoral tunnel, and the distal 2 cm is pulled into the distal tibial tunnel (Fig 10). Therefore, the surgical approach adopted for the control group does not necessitate passage of the entire graft through the tibial tunnel, but only requires measurement of the maximum diameter of the distal 2 cm of the graft. Prior to measurement, the distal 2 cm of the graft was inserted into the bone tunnel. Subsequently, the peak pulling force during retrograde pulling of the graft was measured (Fig 11). The purpose of inserting the distal 2 cm of the graft into the bone tunnel before measurement was to minimize measurement errors caused by the retrograde movement.

### 2.6 Observational indicators

According to the formula for sliding friction (f = μN), a closer match between graft diameter and bone-tunnel diameter will lead to a greater normal force (N) exerted by the tendon on the bone-tunnel interior and a greater sliding friction force (f). Given that the pullout force of the graft from the bone tunnel is directly proportional to f, it can be deduced that a closer graft-tunnel match will lead to a higher value for the measured pullout force.

All grafts used in the present study had diameters of 6–10 mm, and all experiments were conducted by the authors. A total of 35 grafts were separately tested using traditional surgical instruments and modified instruments as follows: (1) Traditional group-the proximal and distal graft diameters, proximal and distal bone-tunnel diameters, peak pulling force for pulling the proximal 2 cm of the graft into the proximal bone tunnel, and peak pulling force for pulling the distal 2 cm of the graft into the distal bone tunnel were separately measured and recorded. (2) Modified group-the proximal and distal graft diameters, proximal and distal bone-tunnel diameters, peak pulling force for pulling the proximal 2 cm of the graft into the proximal bone tunnel, and peak pulling force for pulling the distal 2 cm of the graft into the distal bone tunnel were separately measured and recorded.

### 2.7 Statistical analysis

Traditional group measurement results refer to those of the traditional group, while modified group measurement results pertain to the modified group. Data from both groups were statistically analyzed. Statistical analyses were performed using SPSS 23.0. Measurement data following a normal distribution were expressed as mean ± standard deviation (s), with intergroup comparisons performed using the t-test, and intragroup comparisons performed with the paired t-test. Non-normally distributed data were expressed as median (M) and interquartile ranges (P25, P75), with intergroup comparisons performed using the Mann-Whitney U test and intragroup comparisons performed with the Wilcoxon signed-rank test. Differences were considered statistically significant when P < 0.05.

## 3 Results

### 3.1 Normality test

From Table 1, it can be observed that in the traditional group, the sample sizes for the research data are all less than or equal to 50, warranting the use of the Shapiro-Wilk test. Specifically, the proximal end of the graft, distal end of the graft, proximal diameter of the bone tunnel, distal diameter of the bone tunnel, proximal tendon tension, and distal tendon tension all exhibited significance (p < 0.05), indicating rejection of the null hypothesis (H0: data follows a normal distribution). Thus, in the traditional group, none of the variables including the proximal end of the graft, distal end of the graft, proximal diameter of the bone tunnel, distal diameter of the bone tunnel, proximal tendon tension, and distal tendon tension demonstrate normality.

**Table 1:** The result of the normality test analysis (for the traditional group).

Name	Sample Size	Mean	Standard Deviation	Skewness	Kurtosis	Kolmogorov-Smirnov Test	P	Shapiro-Wilk Test	P
						Statistical Value D		Statistical Value W	
Proximal end of the graft (mm)	35	7.143	0.912	-0.050	-1.498	0.195	0.002**	0.866	0.001**
Distal end of the graft (mm)	35	7.700	0.709	-0.638	0.295	0.178	0.006**	0.926	0.022*
Proximal diameter of the bone tunnel (mm)	35	7.371	1.031	-0.145	-1.239	0.272	0.000**	0.839	0.000**
Distal diameter of the bone tunnel (mm)	35	7.943	0.802	-0.619	0.377	0.300	0.000**	0.834	0.000**
Proximal tendon tension (N)	35	8.591	7.460	1.609	2.197	0.244	0.000**	0.809	0.000**
Distal tendon tension (N)	35	9.140	12.794	4.253	21.484	0.252	0.000**	0.541	0.000**

Note: * *p*<0.05

** *p*<0.01.

From Table 2, it is evident that in the improved group, the sample sizes for the research data are all less than or equal to 50, warranting the use of the Shapiro-Wilk test. Specifically, the proximal end of the graft, proximal diameter of the bone tunnel, proximal tendon tension, and distal tendon tension, a total of 4 variables, exhibited significance (p < 0.05), indicating that the proximal end of the graft, proximal diameter of the bone tunnel, proximal tendon tension, and distal tendon tension do not demonstrate normality. Additionally, the distal end of the graft and distal diameter of the bone tunnel, a total of 2 variables, did not show significance (p > 0.05), implying that the distal end of the graft and distal diameter of the bone tunnel exhibit normality.

**Table 2:** The normality test analysis results (for the modified group).

Name	Sample Size	Mean	Standard Deviation	Skewness	Kurtosis	Kolmogorov-Smirnov Test	P	Shapiro-Wilk Test	P
						Statistical Value D		Statistical Value W	
Proximal end of the graft (mm)	35	7.046	0.822	0.002	-1.530	0.135	0.110	0.893	0.003**
Distal end of the graft (mm)	35	7.117	0.825	0.390	-0.666	0.156	0.030*	0.945	0.080
Proximal diameter of the bone tunnel (mm)	35	7.046	0.822	0.002	-1.530	0.135	0.110	0.893	0.003**
Distal diameter of the bone tunnel (mm)	35	7.117	0.825	0.390	-0.666	0.156	0.030*	0.945	0.080
Proximal tendon tension (N)	35	17.597	18.864	2.043	3.910	0.237	0.000**	0.731	0.000**
Distal tendon tension (N)	35	32.266	25.737	0.622	-1.048	0.169	0.013*	0.884	0.001**

Note: * *p*<0.05

** *p*<0.01.

### 3.2 Comparison of proximal- and distal-graft measurements between the traditional and modified group

A comparison of proximal and distal graft diameter measurements between the traditional and modified groups using the Wilcoxon signed-rank test revealed significant differences between the two paired data sets (p < 0.05). The results are presented in Table 3. Upon detailed analysis, we found that the difference between the traditional and modified groups in proximal graft diameter measurements was significant at the 0.01 level (p < 0.01), with the proximal graft diameter of the traditional group (7.500) being significantly larger than that of the modified group (7.200).

**Table 3:** Comparison of proximal- and distal-graft measurements between the traditional and modified groups.

Name	Median of paired data, M (P25, P75)		Difference in median (M)	*z*	*P*
	Traditional group	Modified group			
Proximal graft (mm)	7.500(6.0,8.0)	7.200(6.2,7.8)	0.300	2.664	0.008**
Distal graft (mm)	8.000(7.5,8.0)	7.000(6.6,7.7)	1.000	4.235	0.000**

Note: * *p*<0.05

** *p*<0.01.

The difference between the traditional and modified groups in distal graft diameter measurements was also significant at the 0.01 level (p < 0.01), with the distal graft diameter of the traditional group (8.000) being significantly larger than that of the modified group (7.000).

### 3.3 Comparison of proximal and distal bone-tunnel diameter measurements between the traditional and modified groups

A comparison of proximal and distal bone-tunnel diameter measurements between the traditional and modified groups using the Wilcoxon signed-rank test revealed significant differences between the two paired data sets (p < 0.05). The results are presented in Table 4. Upon detailed analysis, we found that the difference in proximal bone-tunnel diameter measurement between the traditional and modified groups was significant at the 0.01 level (p < 0.01), with the proximal bone-tunnel diameter of the traditional group (8.000) being significantly larger than that of the modified group (7.200).

**Table 4:** Comparison of proximal and distal bone-tunnel diameter measurements between the traditional and modified groups.

Name	Median of paired data, M (P25, P75)		Difference in median (M)	*z*	*P*
	Traditional group	Modified group			
Proximal-bone- tunnel diameter (mm)	8.000(6.0,8.0)	7.200(6.2,7.8)	0.800	4.003	0.000**
Distal-bone-tunnel diameter (mm)	8.000(8.0,8.0)	7.000(6.6,7.7)	1.000	4.549	0.000**

Note: * *p*<0.05

** *p*<0.01.

The difference in distal bone-tunnel diameter measurement between the traditional and modified groups was also significant at the 0.01 level (p < 0.01), with the distal bone-tunnel diameter of the traditional group (8.000) being significantly larger than that of the modified group (7.000).

### 3.4 Comparison of proximal and distal tendon pulling-force measurements between the traditional and modified groups

A comparison of the proximal and distal tendon pulling-force measurements between the traditional and modified groups using the Wilcoxon signed-rank test revealed significant differences between the two paired data sets (p < 0.05). The results are presented in Table 5. Upon detailed analysis, we found that the difference in proximal tendon pulling-force measurements between the traditional and modified groups was significant at the 0.01 level (p < 0.01), with the proximal tendon pulling force of the traditional group (6.000) being significantly smaller than that of the modified group (9.100).

**Table 5:** Comparison of proximal and distal tendon pulling-force measurements between the traditional and modified groups.

Name	Median of paired data, M (P_25_, P_75_)		Difference in median (M)	*z*	*P*
	Traditional group	Modified group			
Proximal tendon pulling force (N)	6.000(3.9,9.3)	9.100(5.8,20.1)	-3.1	3.836	0.000**
Distal tendon pulling force (N)	6.800(2.5,9.8)	23.900(9.8,54.3)	-17.1	4.468	0.000**

Note: * *p*<0.05

** *p*<0.01.

The difference in distal tendon pulling-force measurements between the traditional and modified groups was also significant at the 0.01 level (p < 0.01), with the distal tendon pulling-force of the traditional group (6.800) being significantly smaller than that of the modified group (23.900).

### 3.5 Comparison between proximal and distal graft diameters, bone-tunnel diameters, and tendon pulling-force measurements after using the modified instruments

When the proximal and distal measurements using the modified instruments were compared by a non-parametric test, we found significant differences in graft diameters, bone-tunnel diameters, and tendon-pulling forces. The results arepresented in Table 6. Analyses using the Mann-Whitney test indicated graft-diameter differences, bone-tunnel diameter differences, and tendon pulling-force differences between the proximal and distal measurements. Upon detailed analysis, we found that when using the modified instruments, the proximal and distal graft diameter measurements were significantly different at the 0.01 level (p < 0.01). A comparison of the median differences showed that the proximal graft diameter measurement (0.000) was significantly smaller than that of the distal graft diameter measurement (-0.500).

**Table 6:** Comparison between proximal and distal graft diameters, bone-tunnel diameters, and tendon pulling-force measurements after using the modified instruments.

Name	Median of proximal/distal data, M (P25, P75)		*z*	*P*
	Proximal end (*n* = 35)	Distal end (*n* = 35)		
Difference in graft diameter (mm)	0.000(-0.3,0.0)	-0.500(-1.0,0.0)	-3.154	0.002**
Difference in bone- tunnel diameter (mm)	-0.200(-0.6,0.0)	-0.800(-1.4,-0.2)	-3.23	0.001**
Difference in tendon- pulling force (N)	4.200(0.0,10.5)	15.400(0.0,44.3)	-2.099	0.036*

Note: * *p*<0.05

** *p*<0.01.

The modified instrument effect on proximal and distal bone-tunnel diameter measurements was also significant at the 0.01 level (p < 0.01). A comparison of the median differences showed that the proximal bone-tunnel diameter measurement (-0.200) was significantly smaller than that of distal bone-tunnel diameter measurement (-0.800).

The modified instrument effect on tendon-pulling force was significant, and revealed a difference at the 0.05 level (p < 0.05) when the proximal and distal measurements were compared. Comparison of the median differences showed that the proximal tendon-pulling force measurement (4.200) was significantly smaller than that of the distal tendon-pulling force measurement (15.400).

## 4 Discussion

The key findings of this study were as follows: (1) The influence of errors in surgical instruments and methods during ligament reconstruction surgery on tunnel widening was investigated for the first time. Although errors are inevitable in all measurements and techniques [21], the impact of such errors on clinical practice should nevertheless be explored and alleviated. The influence of traditional surgical instruments and methods on tunnel widening has been documented in previous literature [1,8,9,22], but further research and improvement in this area has not been reported. (2) The measuring gauge developed by our research group, which is capable of accurate measurement of graft diameter, may mitigate errors arising from graft diameter measurement using traditional measuring cylinders.

The influence of ligament reconstruction surgery on tunnel widening has been studied for years, but the exact cause remains unclear. Tunnel widening usually occurs within 6 months postoperatively and may persist for 1–2 years [7,9,23–25]. This phenomenon arises as a result of multiple types of factors including mechanical, biomechanical, and biological. Specifically, mechanical factors include tunnel location, fixation method, graft selection, graft motion, surgical methods, and rehabilitation methods. Biomechanical factors include foreign-body reaction and thermal necrosis during tunnel creation, and biological factors include tendon-bone healing at the graft-tunnel interface, inflammatory responses induced by increased cytokine production, and infection [1,11]. Researchers have attempted to address the causes of tunnel widening by making various enhancements to surgical methods. These include a shift from fixation of popliteus tendon grafts to press-fitting of patellar tendon grafts, and improvements to press-fitting techniques, graft materials, fixation screw materials, and fixation locations [2,6–8,10,26–30]. Such improvements have achieved certain results but remain incapable of completely eliminating the tunnel-widening phenomenon.

The bungee effect and windshield-wiper effect, which occur after knee ligament reconstruction surgery, are the primary mechanical factors that cause tunnel widening. Both the bungee and windshield-wiper effects maybe caused by graft-tunnel mismatch and instability in graft fixation. In ligament reconstruction surgery, the reduction of bone-tunnel diameter or increase of graft diameter to achieve a snug graft-tunnel fit can provide a more stable biological and mechanical environment, which may help alleviate the bungee and windshield-wiper effects. The wiper effect is the excessive swinging of the graft in the bone marrow canal like a car wiper, resulting in a wiper effect. The bungee effect is that the graft does not have enough initial tension or is not firmly fixed, and the bone marrow canal is too large, causing the graft to relax and tighten in the bone tunnel like a bungee cord. The wiper effect and bungee effect can further enlarge the bone marrow canal, wear and relax the graft, affect the effect of ligament reconstruction, affect the second revision of the ligament, and even lead to the failure of the operation.

**Fig 12:**
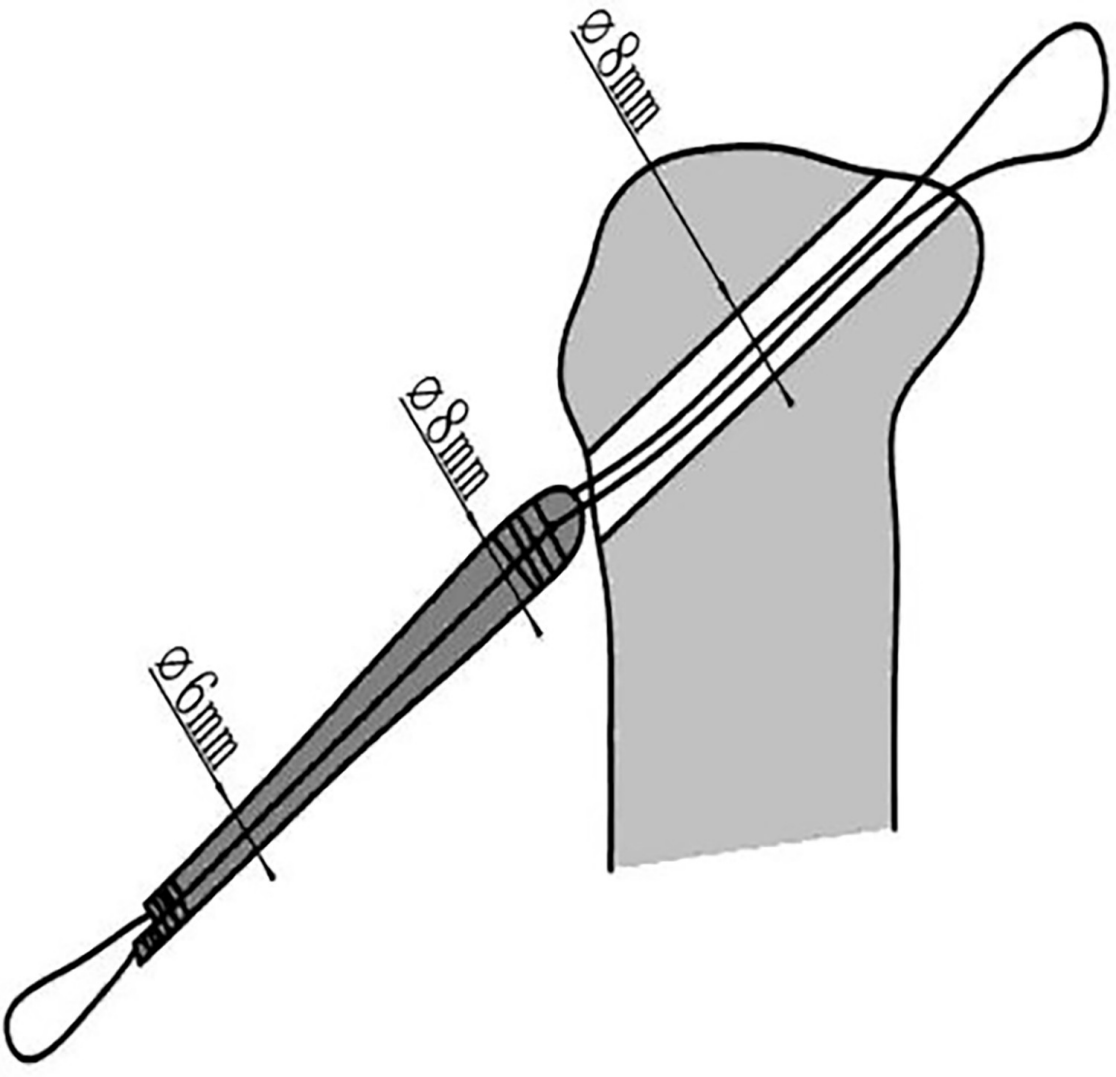
When the proximal graft diameter > distal graft diameter, the tibial-tunnel diameter was taken as the maximum graft diameter.

**Fig 13:**
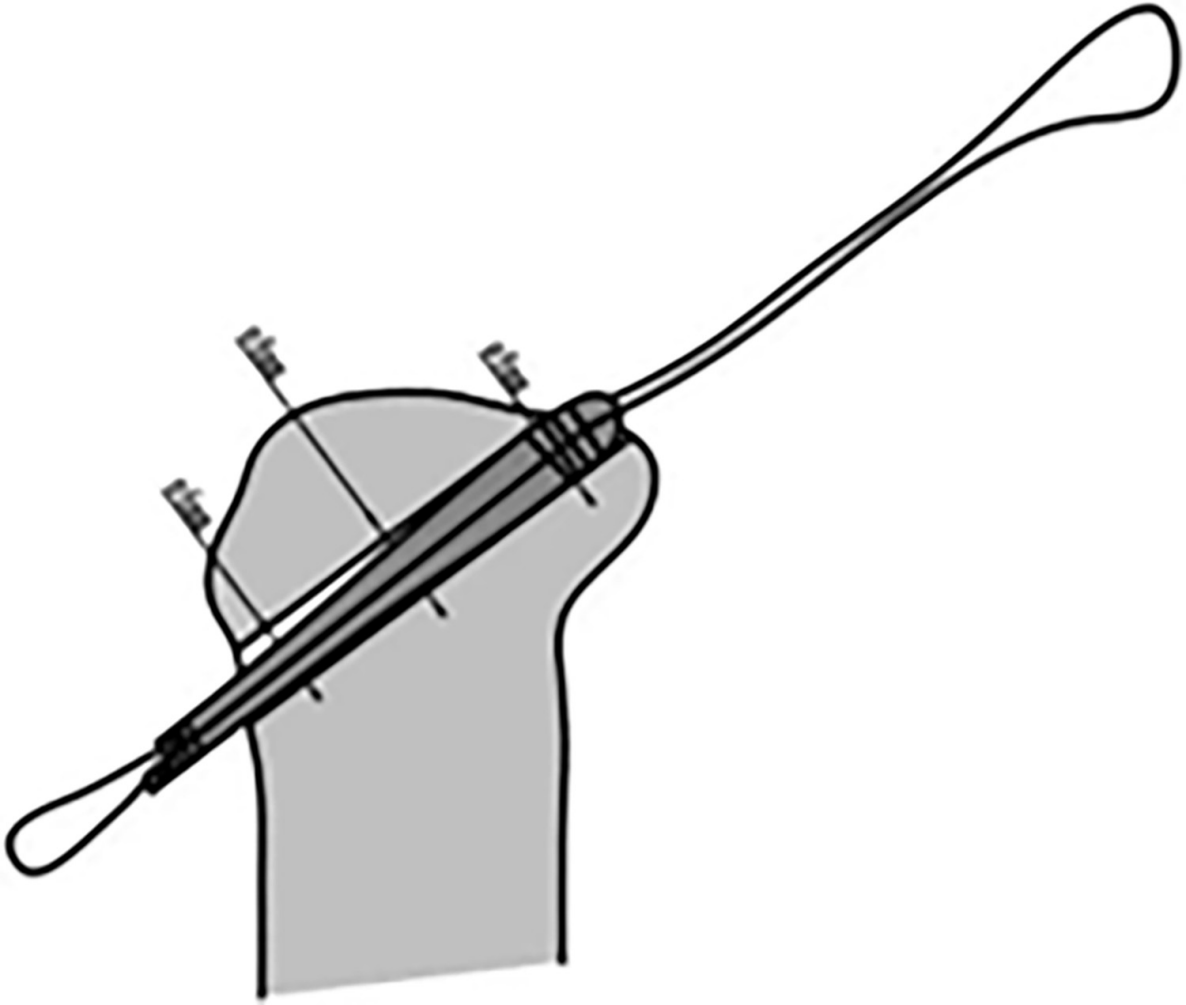
When the proximal graft diameter > distal graft diameter, an obvious graft-tunnel mismatch could be observed after the distal end of the graft had been pulled into the tibial tunnel.

Previous research has indicated that tunnel widening does not affect the short-term clinical effects of anterior cruciate ligament (ACL) reconstruction surgery [1,11,22]. Therefore, physicians may neglect graft-tunnel press-fitting issues while attempting to achieve successful completion of surgical procedures. Limitations in current surgical instruments and methods have caused an increase in graft-tunnel mismatch issues during clinical surgeries. These include the following: (1) Graft diameter measurements obtained using measuring cylinders are estimated values and lack accuracy. Surgical operators often adopt a diameter measurement that is slightly larger than the actual graft diameter to ensure smooth graft passage through the bone tunnel. Such a measurement error leads to a larger bone-tunnel diameter than the graft diameter, thereby inducing graft-tunnel mismatch. (2) The mismatch in the distal graft is larger than that of the proximal graft due to the fact that the distal graft diameter measured by the measuring cylinder is the maximum diameter of the entire graft. For grafts whose proximal diameter is larger than the distal diameter, the error in distal measurements will be more pronounced (Figs 12 and 13). (3) Drill bits used in surgery are only available in diameters of 6 mm, 7 mm, 8 mm, and 9 mm with integer diameter intervals. Given the lack of drill bits with greater precision and degree of match, drill bits with integer diameter values larger than the measured graft diameters are often employed in surgery, which may increase graft-tunnel mismatch. Therefore, the use of the surgical instruments described above may possibly increase the degree of graft-tunnel mismatch, leading to the occurrence of the bungee and windshield-wiper effects, ultimately resulting in tunnel widening.

In the present study, we performed a comparative study of measurements using a self-designed measuring gauge and custom-made drill bits with those using traditional measuring cylinders and drill bits. The results of our comparative experiment revealed that the error arising from measurement of graft diameters with the measuring cylinder was larger than that caused by measurement using the self-developed measuring gauge. The lack of precision drill bits also increases graft-tunnel mismatches. We found that the graft pulling force required for bone tunnels created using the custom-made drill bits was considerably larger than the graft pulling force of the traditional group. Therefore, the experimental results demonstrated that surgical errors and limitations in current surgical instruments and methods contribute to an increase in graft-tunnel mismatch. However, this article still has some limitations. Custom-made drill bits were used in the study. However, drill bits with precise sizes that are compatible with the measurement gauge have not yet been developed. Errors may exist between data obtained from in vitro models and clinical surgical data. Therefore, further validation through clinical surgeries and postoperative bone-tunnel measurements will be required. Retrograde pulling of the graft into the bone tunnel may have caused measurement errors. The measuring gauge may not be positioned at the points of maximum distal or proximal graft diameters during measurement. This may have introduced errors into the measured values. In conclusion, errors during surgery and the limitations of current surgical instruments and methods increase graft-tunnel mismatches, with the degree of mismatch being greater at the tibial end than at the femoral end.
